# IL-7Rα signaling potentiates the anti-tumor activity of NK92 cells

**DOI:** 10.3389/fimmu.2026.1768539

**Published:** 2026-03-30

**Authors:** Chunli Wang, Seokmin Kim, Ling-Zu Kong, Inhwan Jang, Seona Jo, Sunyoung Lee, Soo Yun Lee, Kee K. Kim, Tae-Don Kim

**Affiliations:** 1 Center for Gene and Cell Therapy, Korea Research Institute of Bioscience and Biotechnology (KRIBB), Daejeon, Republic of Korea; 2 Key Laboratory of Laboratory Medicine, Ministry of Education, School of Laboratory Medicine and Life Sciences, Wenzhou Medical University, Wenzhou, Zhejiang, China; 3 Department of Pharmacy, Yonsei Institute of Pharmaceutical Sciences, and Department of Integrative Biotechnology, College of Pharmacy, Yonsei University, Incheon, Republic of Korea; 4 Department of Biochemistry, College of Natural Sciences, Chungnam National University, Daejeon, Republic of Korea; 5 KRIBB School of Advanced Bioconvergence, University of Science and Technology (UST), Daejeon, Republic of Korea

**Keywords:** anticancer therapy, IL-7Ra, immunotherapy, NK cell, receptor engineering

## Abstract

**Background:**

Natural killer (NK) cells are promising candidates for cancer immunotherapy due to their safety and potent anti-tumor activity. However, their therapeutic efficacy is often limited by poor persistence and activity within the tumor microenvironment (TME) caused by a lack of essential cytokines.

**Methods:**

To overcome cytokine dependence, we engineered NK92, primary NK (pNK), and chimeric antigen receptor (CAR)-NK92 cells to express an interleukin-7 receptor with an insertion mutation (IL-7R-IM), which induces constitutive signaling. We evaluated the proliferation, viability, and cytotoxicity of these cells *in vitro* and analyzed downstream signaling pathways using RNA sequencing and western blotting. The *in vivo* anti-tumor efficacy was assessed using a metastatic leukemia xenograft mouse model.

**Results:**

NK92-IL-7R-IM cells exhibited sustained proliferation and high viability independent of exogenous cytokines, superior to IL-2-activated NK cells. This enhanced functionality was driven by the constitutive activation of the JAK/STAT, AKT, and ERK signaling pathways, leading to the upregulation of cytotoxicity-related genes (*GZMA*, *GZMB*) and anti-apoptotic genes (*BCL2L1*). *In vivo*, NK92-IL-7R-IM cells demonstrated significantly potent anti-tumor activity and extended survival compared to control groups. Furthermore, the IL-7R-IM strategy successfully enhanced the function of CAR-NK cells targeting EphA2, EGFR, and CD5 antigens.

**Conclusions:**

The expression of IL-7R-IM confers cytokine independence and robust anti-tumor activity to NK and CAR-NK cells. This strategy offers a practical solution to improve the persistence and efficacy of off-the-shelf NK cell therapeutics for clinical application.

## Introduction

Natural killer (NK) cells are innate lymphoid cells which are capable of recognizing and eliminating tumor cells, and they play pivotal roles in both anti-viral and anti-tumor immunity (1, 2). NK cells rely various receptor types to recognize tumor cells, and have little or no sensitivity for foreign major histocompatibility complex class I receptors (3). Therefore, NK cells pose minimal risks of inducing graft-versus-host disease and can be used in an allogeneic setting, making them important tools in immunotherapy (4–6). Among the various types of NK cell-based anti-tumor immunotherapies, chimeric antigen receptor (CAR)-NK cells are now recognized as a promising anti-cancer therapeutic strategy (7, 8). NK92 cells are cytokine-dependent immortalized cells that are widely used in immunotherapy research and applications (9–11). However, NK and CAR-NK cells have poor activity in the absence of cytokines, which affect the clinical effectiveness of NK cell therapy (10, 12). Therefore, successful NK cell therapy requires cytokine co-administration, which is a disadvantage.

Members of the type I cytokine family, including interleukin-2 (IL-2), play vital roles in innate and active immune homeostasis (13). These cytokines are harnessed to restore compromised NK cell functionality within the tumor microenvironment (2, 14, 15). IL-2 is the most widely researched cytokine activator of NK cells. Currently, exogenous IL-2 is injected into patients with tumors to increase the cytotoxic activity of NK cells (16–18). However, IL-2 has been found to activate bystander immune cells, such as regulatory T cells (Tregs), leading to severe toxicity (19–21). In addition, IL-2 activated Tregs can also inhibit the anti-tumor activity of NK cells (19, 22). Recently, two novel types of non-secretory fusion protein IL-2 have been developed: endoplasmic reticulum-retained IL-2 (10) and membrane-bound IL-2 (12), which address the problems of IL-2 secretion and bystander cell activation. However, the former is still limited by the number and affinity of the intracellular IL-2Rα receptors. The latter induces IL-2 fusion with IL-2R to bypass challenges related to poor receptor affinity, but the detailed mechanism is unclear. Thus, there remains a need for novel strategies which enhance the viability and cytotoxic activity of NK cells in the tumor microenvironment, while reducing their side effects.

Current studies on IL-7 have mainly focused on enhancing the proliferation, survival, and persistence of T cells by activating the IL-7 receptor (IL-7R) (23). Recombinant long-acting IL-7 (rhIL-7-hyFc) has been used in T- and CAR-T cell research and has demonstrated good cytotoxicity and persistence (24, 25). Furthermore, a recent study demonstrated that rhIL-7-hyFc significantly prolonged survival and reduced tumor burden in BCMA CAR-iNKT-treated mice (26). The few existing studies have shown that IL-7 enhances IFN-γ secretion and NK cell survival (27, 28), and that its receptor (IL-7R) is expressed at relatively low levels in NK cells (29). Notably, gain-of-function insertion mutations (IM) in the IL-7R transmembrane domain were originally identified as oncogenic drivers in T-cell acute lymphoblastic leukemia (T-ALL), conferring constitutive receptor activation independent of ligand binding (30, 31). However, we hypothesized that this constitutive signaling could be repurposed to enhance NK cell fitness without driving malignancy. In this study, we propose that IL-7R-IM signaling primarily mediates essential proliferative and cytotoxic responses in NK cells rather than inducing malignant transformation. This is supported by the observation that these engineered cells do not exhibit uncontrolled long-term expansion *in vivo*, aligning with the safety profile required for therapy. Therefore, we focused on using signaling molecules downstream of IL-7R to improve the viability and anti-tumor activity of NK92 and primary NK cells under cytokine-independent conditions and demonstrated the underlying mechanisms of IL-7R-IM signaling in NK cells and its potential clinical implications.

Herein, we evaluated the effects of IL-7R and IL-7R-IM on the proliferation, viability, tumor-killing activity, and cytokine production of NK92 and primary NK cells, and further explored the molecular mechanisms underlying IL-7R-IM function. In addition, we investigated the role of IL-7R-IM in three types of CAR-NK92 cells. Our results show that NK92-IL-7R-IM and CAR-NK92-IL-7R-IM cells exhibit higher cytotoxicity and cell viability *in vitro*, NK92-IL-7R-IM had higher anti-tumor activity *in vivo* than normal NK cells, without the addition of exogenous cytokines. In conclusion, expression of IL-7R-IM in NK or CAR-NK cells is an effective strategy for improving NK cell immunotherapy.

## Methods and materials

### Cell culture

NK92 (human natural killer cell line, ATCC®CRL-2407™), MDA-MB-231 (Human breast cancer cell line, ATCC^®^CRM-HTB-26™), NCI-H460 (human non-small cell lung cancer, ATCC^®^HTB-177™), MOLT 4 (human T lymphoblast cell line, ATCC^®^CRL-1582™), K562 (human leukemia cell line, ATCC®CCL-243™), and HEK-293T cells (ATCC^®^CRL-11268™) were acquired from the American Type Culture Collection (Manassas, VA, USA). EphA2-CAR-NK92, CD5-CAR-NK92, and ZEGFR-CAR-NK92 cell lines were generated within our laboratory (unpublished data). NK92 cells were maintained in ATCC-recommended medium. The concentration of IL-2 in all experimental treatments was 20 ng/mL. All cancer cells were cultured in RPMI 1640 medium supplemented with 10% heat-inactivated fetal bovine serum (FBS; Seradigm, USA) and 1% streptomycin/penicillin (Gibco, USA). HEK-293T cells were cultured in DMEM supplemented with 1% penicillin/streptomycin and 10% FBS.

### Isolation and culture of primary human NK cells

Primary human CD56+CD3- NK cells were isolated from umbilical cord blood samples from healthy donors, which was approved by the Public Institutional Review Board designated by the Ministry of Health and Welfare (IRB approval number: P01-201610-31-002). RosetteSep™ Human CD3 Depletion Cocktail and Ficoll density gradient centrifugation were performed to collect mononuclear cells (MNC). MNC was washed with wash buffer (1 X PBS and 2% FBS), centrifuged to remove the supernatant, and treated with preheated ACK to remove any red blood cells. Finally, MNC was washed with wash buffer before being activated and expanded in alpha minimum essential medium, which contained 10% FBS, 10 ng/ mL IL-21, 10 ng/mL IL-15, 1 μM hydrocortisone, and 1% penicillin/streptomycin. NK cell purity was determined using a BD FACS Canto II cytometer (BD Biosciences, Franklin Lakes, NK, USA) on days 0, 5, and 9 using fluorochrome-conjugated antibodies against CD3 and CD56 (BD Biosciences, San Jose, CA, USA). Cells demonstrating a CD56+ fraction of more than 85% (indicating NK cells) were used for further experiments.

### Generation of lentiviral vectors and lentivirus infection cell lines

The coding sequence (CDS) of IL-7R-WT was amplified through PCR using primers F and R from a Human fibroblast cDNA library (**Supplementary Table S1**). The IL-7R-WT CDS sequence was used as a template to amplify the IL-7R-IM CDS via overlapping extended PCR with primers F, R1, F1, and R (**Supplementary Table S1**). The PCR products were restriction enzyme digested and ligated into the corresponding restriction sites of the pLVX-EF1α-IRES-ZsGreen1 lentiviral vector. NK92-IL-7R-WT cells were generated via transduction with the lentiviral vector (pLVX-EF1α-IL-7R-WT-IRES-ZsGreen1). To generate the lentivirus, the pLVX-EF1α-IRES-ZsGreen1 vector, packaging vector, and enveloping vector were transfected to HEK-293T cells using TransIT-2020 transfection reagents. After 3 days of cell culture, the supernatant was collected and the virus was condensed by ultracentrifugation. NK92-IL-7R-IM, CD5-CAR-IL-7R-IM, EpHA2-CAR-IL-7R-IM, and ZEGFR-CAR-IL-7R-IM cells were also generated using this lentiviral vector (pLVX-EF1α-IL-7R-IM-IRES-ZsGreen1). Finally, NK92 cells were sorted using fluorescence-activated cell sorting (FACS) to obtain positive cells.

### *In vitro* mRNA transcription and generation of IL-7R-WT and IL-7R-IM pNK cells

IL-7R-WT and IL-7R-IM CDS sequences were restriction enzyme digested and ligated into the corresponding restriction sites of the pSK5'-UTR-vector. The IL-7R-WT- and IL-7R-IM-encoding mRNAs were generated using an mMESSAGE mMACHINE® T7 Ultra Kit (Thermo Fisher Science, Waltham, MA, USA) according to the manufacturer’s instructions. Subsequently, the pNK cells were electroporated with either mRNA encoding IL-7R-WT or IL-7R-IM (10 μg mRNA per million cells). Cell expression, cytotoxicity, and viability were detected 12 h after electroporation.

### Flow cytometry analysis

The cells were acquired using cold FACS buffer and labeled with fluorochrome-conjugated antibodies against CD127 (IL-7R, BD Biosciences, San Jose, CA, USA) and Myc-Tag (9B11) (Cell Signaling Technology, Danvers, MA, USA). The antibodies were incubated with the cells for 30 minutes at 4 °C in the dark. For degranulation activity assessments, NK cells and target cells were combined and co-incubated for 4 hours at 37 °C at a constant effector: target (E:T) cell ratio, and then CD107a (BD Biosciences, San Jose, CA, USA) was incubated with the cells for 30 minutes at 4°C in the dark. For apoptosis assessments, cells were treated with Annexin V and propidium iodide according to the BD Apoptosis Detection Kit II instructions. Cells were detected by flow cytometry and the resulting data were analyzed using FlowJo software (BD Biosciences, San Jose, CA, USA).

### Cell proliferation

Cell proliferation assays were conducted using a CCK-8 kit (Dojindo, Mashiki, Japan). 2.0×10^4^ cells per well were initially seeded in 96-well plates with 100 µL of culture medium. At specific time intervals (0, 1, 2, and 3 days), 10 µL of CCK-8 reagent was introduced into each well and incubated at 37 °C for 4 hours. Subsequently, the absorbance at 450 nm was detected utilizing a microplate reader (Molecular Devices, San Jose, CA, USA). Cell numbers were measured via trypan blue staining with automated cell counters (Thermo Fisher Science, Waltham, MA, USA).

### Calcein AM-based cytotoxicity

NK cell cytotoxicity was detected using calcein-AM (C1430, Thermo Fisher, Waltham, MA, USA) as described previously (32). Briefly, target cells were co-incubated with calcein. Subsequently, NK cells and target cells were combined and co-incubated for 4 hours at 37 °C at a constant E:T cell ratio. Supernatant calcein quantification was conducted using a multimode microplate reader (Molecular Devices, San Jose, CA, USA) and computed in accordance with the provided formula.

### ELISA analysis for measurement of IFN-γ and TNF-α

NK92 and target cells were co-cultured for 12 h at 37 °C at the given E:T cell ratios. Cell supernatants were collected and IFN-γ and TNF-α secretion was tested using ELISA kits (n=3)(Invitrogen, Waltham, MA, USA) according to manufacturer's instructions.

### RNA sequencing

#### RNA sequencing sample preparation

Total RNA was isolated from NK92 cells incubated with IL-2 for 2 or 4 days, and NK92-IL-7R-IM cells without cytokines for 4 days. The cells were collected and washed with phosphate-buffered saline (PBS), and total RNA was isolated using TRIzol reagent for RNA-seq library construction and sequencing.

#### Processing of RNA-seq data

Sequencing was outsourced to Macrogen (Republic of Korea) and performed on a NovaSeq6000 platform (Illumina, CA, USA) for transcriptome sequencing of paired-end reads (101 bp). The Ensembl genome browser was used to obtain the reference genome sequence of *Homo sapiens* (assembly ID: GRCh38). HISAT2 software (ver. 2.2.0) was used to index the reference genome and map the sequencing reads of the samples. Subsequently, featureCounts (ver. 2.0.1) software was used to calculate the BAM files generated using SAMtools (ver. 1.15.1).

#### Gene expression analysis

Principal component analysis (PCA) of the differentially expressed genes with standard deviations (STD) > 0.3 was used to visualize the separation between each group of NK cells. We selected the significantly differentially expressed genes (based on a fold change (FC) threshold >1.5 and *p* < 0.01 from Student’s t tests) between NK92 and NK92-IL-7R-IM cells and labeled the top 15 genes that were most upregulated or downregulated in the volcano plot. To characterize each group (IL2-2D, IL-4D, and IM-4D), we selected 3,242 genes with STD > 0.3 and that were statistically significant in the analysis of variance (ANOVA; *p* < 0.01, empirical Bayes probability (EBP) < 0.01) and performed a hierarchical clustering analysis using the centroid linkage method with GeneCluster 3.0. To investigate the significantly enriched functions, we performed biological process of Gene Ontology (GO), Kyoto Encyclopedia of Genes and Genomes (KEGG), and REACTOME pathway enrichment analyses using the DAVID tool (http://david.ncifcrf.gov) with the following significance thresholds: *p* < 0.01, false discovery rate (FDR) < 0.01. R software (ver. 3.6.3) was used for all statistical analyses of gene expression.

### Western blotting

The cells were lysed with a protein lysis buffer, and protease and phosphatase inhibitors (Roche) were added. Approximately 30 µg of total proteins per sample were separated on 12% SDS-PAGE gels and subsequently transferred onto polyvinylidene difluoride membranes. Following this, the membranes were immunoblotted with primary antibodies against protein kinase B (AKT), phosphorylated AKT (p-AKT, S473), extracellular signal-regulated kinase (ERK), p-ERK, signal transduction factor and transcription activator 5 (STAT5), p-STAT5, STAT3, p-STAT3, Janus kinase 1 (JAK1), p-JAK1, p-JAK3, JAK3, and GAPDH (Cell Signaling Technology, Danvers, MA, USA). The membranes were then incubated with horseradish peroxidase-labeled secondary antibodies (Thermo Fisher, Waltham, MA, USA). Finally, protein detection was carried out using an enhanced chemiluminescence plus western blot detection system (Thermo Fisher, Waltham, MA, USA).

### *In vivo* anti-tumor assays

All experimental mice were bred under specific pathogen-free conditions. The animal procedures adhered to the regulations established by the Institutional Animal Care and Use Committee of the Korea Research Institute of Bioscience and Biotechnology (approval ID: KRIBB-AEC 23001). These experiments were carried out in strict compliance with the Institutional Guidelines for Animal Care provided by the National Institutes of Health, Bethesda, MD, USA and used the ARRIVE reporting guidelines (33). For Metastatic leukemia mouse model, male NOD Cg-Prkdcscid Il2rgtm1 Sug/Jic (NOG) mice were purchased from Saeronbio (Republic of Korea). 2 × 10^6^ K562-Luc cells (stored in our laboratory^17^) were injected intravenously (i.v) into 6-week-old male NOG mice. Mice were then injected with 4×10^6^ NK92 (control group) or NK92-IL-7R-IM cells through the tail vein (4 mice per group). NK92 and NK92-IL-7R-IM were not irradiated before injection. Tumor sizes were measured via bioluminescence using an *in vivo* imaging system (IVIS). Mice were stunned with 1-3% isoflurane and intraperitoneally injected with 150 mg/kg of d-luciferin (PerkinElmer). After 10 min, the luciferase activity of the K562-Luc cells was detected using an IVIS (Caliper Life Sciences). Results were analyzed using Living Image software (PerkinElmer).

### Statistical analysis

All experimental findings are presented as the mean ± SEM derived from a minimum of three independent experiments. Student's t-tests and one- or two-way analysis of variance (ANOVA) were employed to evaluate statistical differences among groups. For mouse survival rate analyses, log-rank (Mantel–Cox) tests were applied. GraphPad Prism 7.0 was utilized for all statistical analyses, with significance denoted at *p < 0.05, **p < 0.01.

## Results

### Wild type IL-7R improves the proliferation, viability and function of NK92 cells

**Figure 1:**
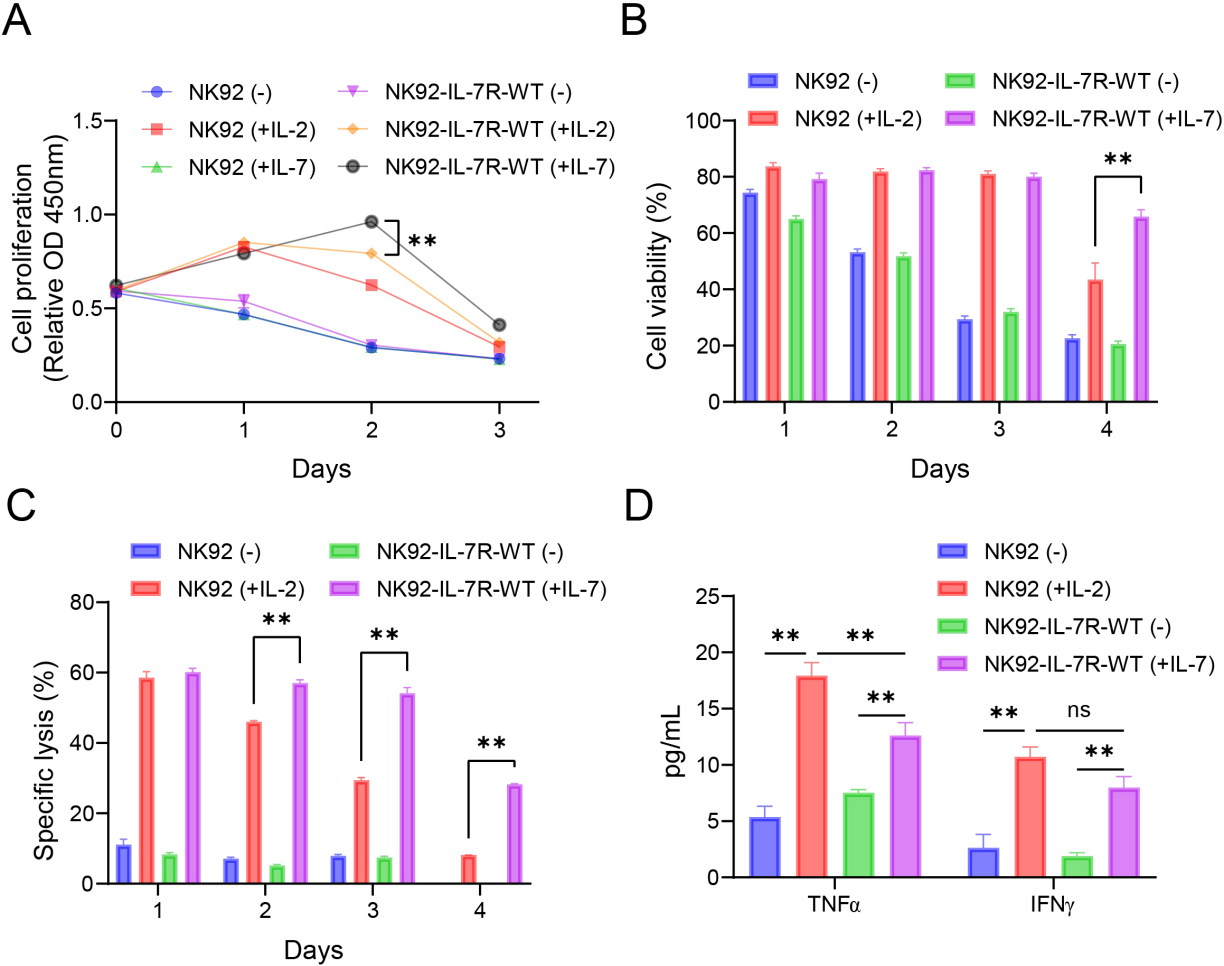
IL-7R-WT promotes the proliferation, viability, and function of NK92 cells **(A)** CCK-8 proliferation assay results of NK92 and NK92-IL-7R-WT cells maintained with IL-2 (20 ng/mL), IL-7 (20 ng/mL), or without cytokines (–) in culture for 1–3 days. **(B)** Cell viabilities of NK92 and NK92-IL-7R-WT cells maintained with IL-2 (20 ng/mL), IL-7 (20 ng/mL), or without cytokines (-). **(C)** Cytotoxicity of NK92 and NK92-IL-7R-WT cells maintained with IL-2 (20 ng/mL), IL-7 (20 ng/mL), or without cytokines (-) against K562 cells; effector: target (E:T) ratio=5:1. **(D)** TNF-α and IFN-γ release as detected by ELISA using the culture medium of cells co-cultured with K562 cells; E :T ratio=5:1. Data represent the mean ± SEM of three independent experimental replicates. Statistical significance analyzed by two-way ANOVA followed by Tukey’s multiple comparisons test. **p < 0.01.

To verify the function of IL-7R in NK92 cells, IL-7R-WT was first amplified from human fibroblasts via PCR, and the pLVX-EF1α-IL-7R-IRES-ZsGreen1 vector was constructed (**Supplementary Figure S1A**). NK92 cells were transduced with IL-7R-WT-carrying lentivirus and sorted by flow cytometry using the ZsGreen1 reporter gene to obtain NK92-IL-7R-WT cells. Finally, IL-7R expression was observed in NK92 cells using flow cytometry (**Supplementary Figure S1B**). Since IL-2 is a key cytokine in activating NK92 cells, NK92 cells maintained with IL-2 (+IL-2–20 ng/mL) were used as a positive control group, and NK92 cells maintained without any cytokines (–) were used as a negative control group to compare the effects of IL-7-IL-7R on NK92 cells. To verify the function of NK92-IL-7R-WT cells, we investigated their proliferative capacity. The Cell Counting Kit-8 (CCK-8) results showed that the NK92-IL-7R-WT (+IL-7–20 ng/mL) group demonstrated significantly higher proliferation than the NK92 (+IL-2-20), (+IL-7-20), and cytokines (–), NK92-IL-7R-WT (+IL-2-20), and cytokines (–) groups from day 2 (**Figure 1A**) (*p* < 0.01), suggesting that IL-7 has a strong role in promoting the proliferation of NK92 cells. Additional cell viability assays showed that the NK92-IL-7R-WT (+IL-7) group had higher cell viability than the NK92 (+IL-2) and (–) groups on day 4 of culture (**Figure 1B**) (*p* < 0.01). Cytokines not only maintain the proliferation and viability of NK92 cells but also enhance their tumor-killing activity. Therefore, we detected the cytolytic activity of NK92 cells against K562 cells. Results showed that the cytotoxicity of the NK92-IL-7R-WT (+IL-7) group was significantly higher than that of the NK92 (+IL-2-20) group from day 2 (**Figure 1C**) (*p* < 0.01). Moreover, the NK92-IL-7R-WT (+IL-7) group released significantly more cytokines (IFN-γ and TNF-α) when compared to the NK92-IL-7R-WT (–) group after co-culture with K562 cells (**Figure 1D**) (*p* < 0.01). These elevated cytokine levels support the promotional effect of IL-7 on the tumor-killing activity of NK92-IL-7R-WT cells. Taken together, these results emphasize that the expression of IL-7R-WT, specifically in the presence of exogenous IL-7, promotes the proliferation, viability, and tumor-killing activity of NK92 cells. Furthermore, the viability of NK92-IL-7R-WT cells stimulated with IL-7 was superior to that of parental NK92 cells treated with IL-2.

### IL-7R-IM promotes the proliferation, viability, and function of NK92 and primary NK cells

**Figure 2:**
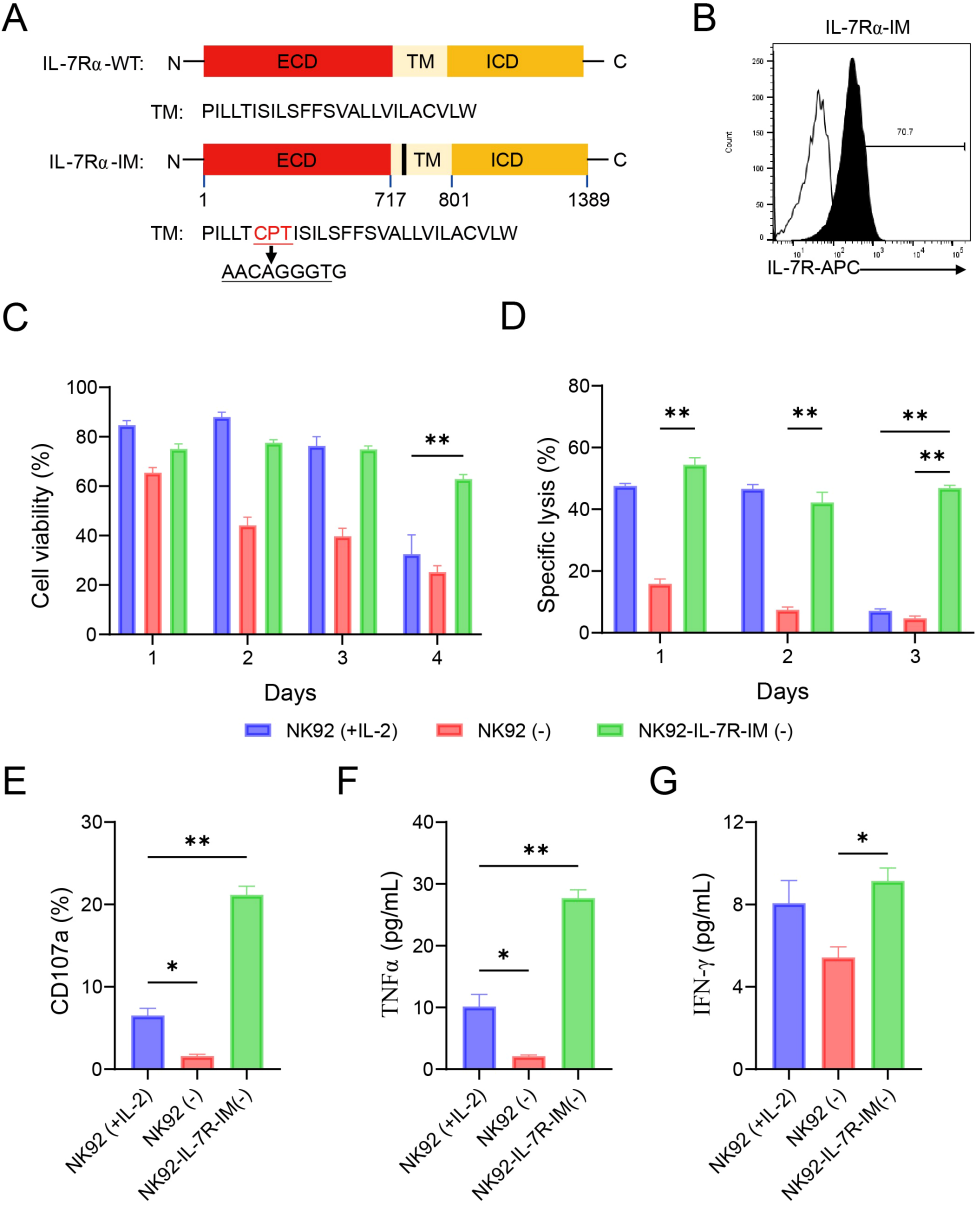
IL-7R-IM promotes the proliferation, viability, and function of NK92 cells **(A)** Schematic representation of the IL-7R insertional mutation (IM) expression cassette. **(B)** Flow cytometry detects showing the expression of IL-7R-IM in NK92 cells. **(C)** Cell viabilities of NK92 (+IL-2), NK92 (-), and NK92-IL-7R-IM (-) cells. **(D)** Cytotoxicity against K562 cells of the three groups of cells after being cultured for 1–3 days; E:T ratio=5:1. **(E)** Degranulation was detected in the cells by flow cytometry after 3 days of culture and 4 h of co-incubation with K562 cells at E:T =5:1. **(F, G)** IFN-γ and TNF-α release as detected by ELISA using the culture medium of the three groups of cells after 3 days of culture and 12 h of co-incubation with K562 cells at E:T =5:1. Data represent the mean ± SEM of three independent experimental replicates. Statistical significance analyzed by two-way ANOVA followed by Tukey’s multiple comparisons test. **(C, D)** Statistical significance was assessed by ordinary one-way ANOVA. **(E-G)** **p < 0.01; *p < 0.05.

To evaluate the viability and cytotoxicity of NK92 cells in the absence of exogenous cytokines, we will explore the constitutively active form of IL-7R on the function of NK92 cells. Firstly, 9 bp was inserted into the IL-7R-WT CDS sequence to obtain the IL-7R-IM sequence (**Figure 2A**). The procedure for obtaining NK92-IL-7R-IM cells was the same as that for producing NK92-IL-7R-WT cells. Finally, IL-7R-IM expression levels in the NK92 cells were detected by flow cytometry (**Figure 2B**). The proliferation of NK92-IL-7R-IM cells can be independent of cytokines (**Supplementary Figure S1C**). We observed that NK92-IL-7R-IM cells cultured in the absence of cytokines demonstrated comparable or slightly enhanced proliferation capacities relative to parental NK92 cells maintained with IL-2 (**Supplementary Figure S1D**) (*p* < 0.01). In terms of viability, cytokine-free NK92-IL-7R-IM cells mirrored the survival profile of IL-2-supplemented NK92 cells during the first 3 days but exhibited significantly superior viability by day 4 (**Figure 2C**) (*p* < 0.01). Furthermore, the cytotoxicity of the NK92-IL-7R-IM cells was significantly higher than that of the cytokine-deprived NK92 controls on days 1-3 (**Figure 2D**) (*p* < 0.01). At day 3, the tumor-killing activity of NK92-IL-7R-IM cells showed a positively correlated with effector cell numbers and was significantly higher than that of IL-2-treated NK92 cells across different effector: target (E:T) cell ratios (**Supplementary Figure S1E**) (*p* < 0.01). Moreover, NK92-IL-7R-IM cells displayed significantly elevated degranulation activity (*p* < 0.01) and TNF-α release (*p* < 0.01) compared to IL-2-treated controls, as well as increased IFN-γ secretion compared to cytokine-deprived parental cells at day 3 (*p* < 0.05) (**Figures 2E, F, G**). Altogether, these results suggested that constitutive IL-7R signaling was more effective than IL-2 co-administration in maintaining NK cell viability and tumor killing activity.

**Figure 3:**
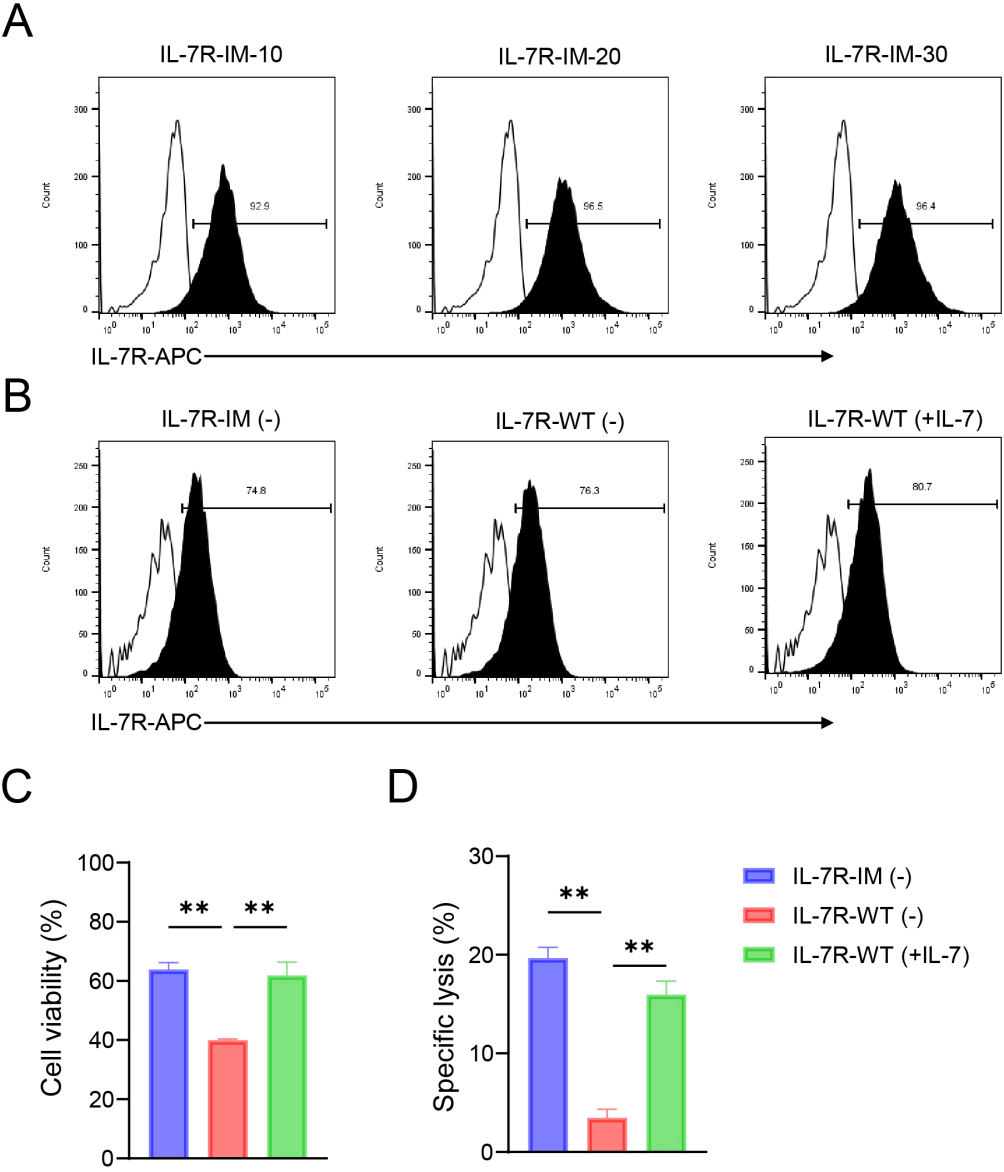
Effects of IL-7R-IM mRNA on cell viability and cytotoxicity of pNK cells **(A)** The expression of IL-7R-IM mRNA with different amount (10, 20, 30 µg) in pNK cells was detected by flow cytometry. **(B)** The expression of IL-7R-IM and IL-7R-WT of 10 µg mRNA in pNK cells was detected by flow cytometry. **(C)** Cell viabilities of the three groups of cells 12 h after electroporation of 10 µg mRNA. **(D)** Cytotoxic activities of the three groups of cells 12h after electroporation of 10 µg mRNA (n=3) E:T = 2:1). Data represent the mean ± SEM of three independent experimental replicates. Statistical significance analyzed by ordinary one-way ANOVA followed by Tukey’s multiple comparisons test. **p < 0.01.

To further verify the function of IL-7R-IM, we constructed mRNA vectors containing IL-7R-WT and IL-7R-IM, with IL-7R-WT serving as a control to compare the expression and activity of the two mRNAs in primary human NK (pNK) cells *in vitro*. We used mRNA electroporation to express IL-7R-IM in pNK cells and determined that the optimal transfection quantity of mRNA was 10 μg (**Figure 3A**), with a transfection efficiency of close to 80% (**Figure 3B**). The same amount of IL-7R-IM and IL-7R-WT mRNA was used to electroporation of pNK cells (n=3) to detect the effect of IL-7R-IM mRNA on the viability and cytotoxicity of pNK cells. Results showed that IL-7R-IM-expressing pNK cells maintained without cytokines possessed similar cell viability and cancer-killing effects as IL-7R-WT-expressing pNK cells maintained with IL-7, and significantly higher cell viability and cytotoxicity than IL-7R-WT-expressing pNK cells maintained without IL-7 (**Figures 3C, D**) (*p* < 0.01). These results suggested that IL-7R-IM has the same function in pNK and NK92 cells and can maintain cell viability and NK cell cytotoxicity in the absence of cytokines.

### IL-7R-IM affects the signal output and gene expression profile of NK92 cells

**Figure 4:**
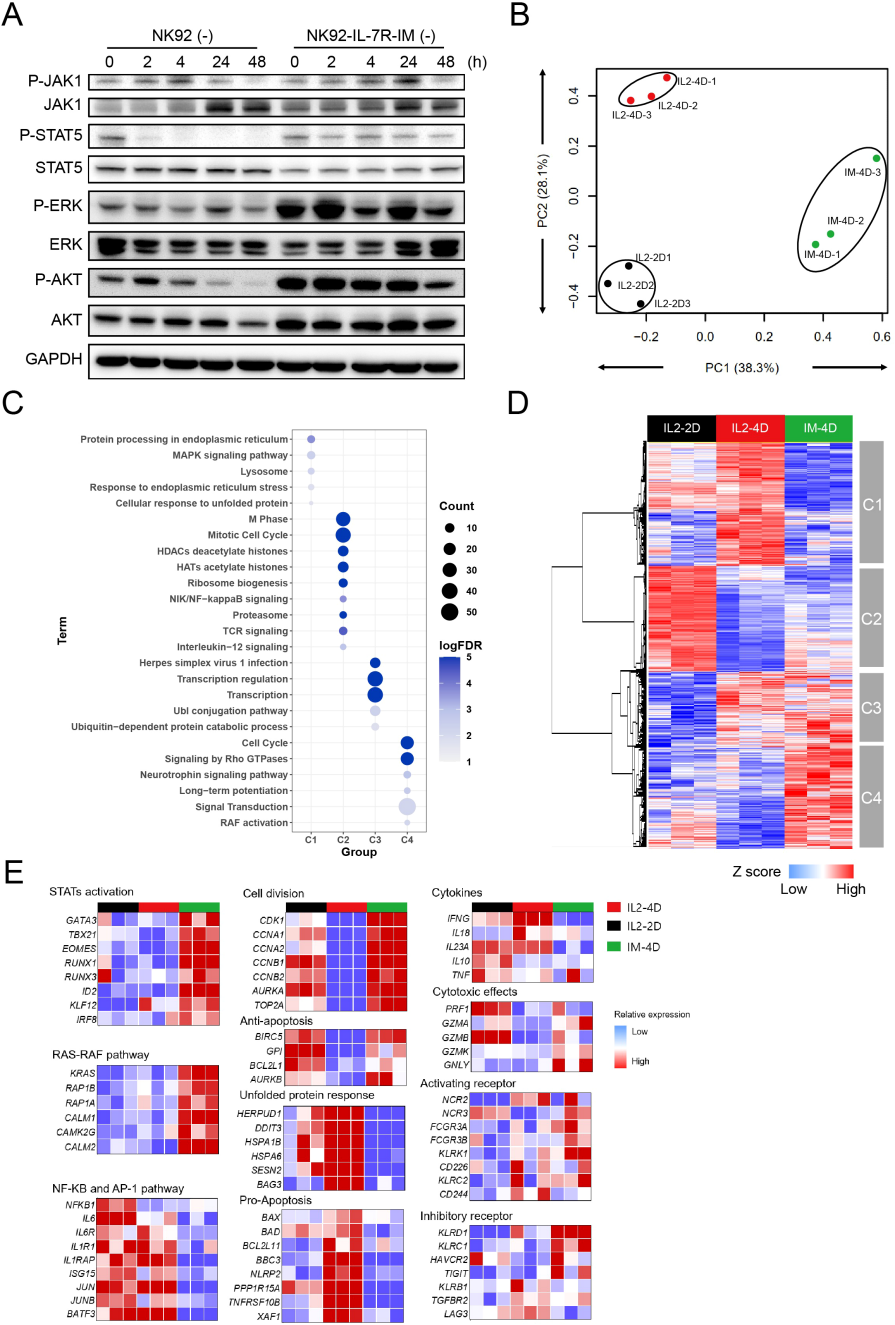
IL-7R-IM affects the gene expression profile of NK92 cells **(A)** Western blot evaluation results of JAK1, STAT5, ERK, and AKT kinase phosphorylation in NK92 and NK92-IL-7R-IM cells maintained without cytokines for 0, 2, 4, 24, and 48 (h) **(B)** PCA results of NK92 and NK92-IL-7R-IM cell transcriptome profiles (n=3). NK92 maintained with IL-2 for 2 days (IL-2-D2), NK92 maintained with IL-2 for 4 days (IL-2-D4), and NK92-IL-7R-IM cells maintained without cytokines for 4 days (IM-4D). **(C)** Dot plot of gene ontology (GO) functional clustering of differentially expressed genes. **(D)** Hierarchical clustering of the IL-2-2D, IL-2-4D, and IM-4D transcriptome profiles. **(E)** The differentially expressed genes in IL-2-2D, IL-2-4D and IM-4D group were divided into categories according to their function. The treatment concentration of IL-2 was 20 ng/mL.

To understand the biological mechanisms by which IL-7R-IM enhances cell viability, cytotoxicity, and cytokine release, we delved into the signaling pathways within NK cells. Cytokines promote NK cell viability and inhibit apoptosis, mainly via the JAK/STAT5, PI3K-AKT, and MEK/ERK signaling pathways (34). We found that NK92-IL-7R-IM cells continuously activated the JAK1/STAT5, ERK, and AKT signaling pathways independently of cytokine stimulation (**Figure 4A**). The additional test showed that the phosphorylation of STAT3 and STAT5, as well as AKT and ERK, in NK92-IL-7R-IM lysates, remains sustained over time even in the absence of cytokines (**Supplementary Figure S1F**). These results suggest that IL-7R-IM can be continuously activated to maintain cell viability and tumor-killing capacity, which is consistent with previous studies (35).

To further determine the differences in downstream signaling in IL-7- and IL-2-activated NK cells, we compared the gene expression profiles of NK92 cells maintained with IL-2 for 2 days (IL-2-2D) or 4 days (IL-2-4D), and NK92-IL-7R-IM cells maintained without cytokines for 4 days (IM-4D). The two-dimensional distribution projected by principal component analysis (PCA) revealed a clear separation among the three groups, indicating that the IM-4D group was biologically distinct from the IL-2-2D and IL-2-4D groups (**Figure 4B**). To compare differentially expressed genes between the IM-4D, IL-2-2D, and IL-2-4D groups, we performed volcano analysis using a threshold fold-change > 1.5 and *p* < 0.01. Compared to the IL-2-2D and IL-2-4D groups, 959 differentially expressed genes were identified in the IL-7R-IM group, which included 202 downregulated genes and 747 upregulated genes (**Supplementary Figure S2**). The top 15 differentially expressed upregulated and downregulated genes are shown in **Supplementary Figure S2**. We found that upregulated RNF125, CDH13, and CD52 were closely related to T-cell receptor signaling (36), cell growth and survival (37), and immune regulation (38), respectively, indicating that IL-7R-IM plays an important role in regulating cell signaling and survival (**Supplementary Figure S2**).

To further understand the molecular mechanisms underlying the functional differences between IL-7R-IM and IL-2 on NK92 cells, hierarchical clustering analysis and gene ontology (GO) enrichment analysis was performed. The results showed that key cell growth processes (indicated by increased expression levels of cell cycle and RAS-RAF pathway-related genes) were upregulated in the IL-7R-IM group when compared with the IL-2-4D group. Moreover, key cell damage processes such as stress response and apoptosis were downregulated in the IL-7R-IM group (**Figures 4C, D**) (*p* < 0.01). In addition, to further determine the mechanism by which IL-7R-IM affects NK92 cell function, we analyzed the changes in the expression levels of relevant activating and inhibitory receptors, signaling pathways, apoptosis-related genes, and cytotoxicity-related genes in NK92 cells. When compared to the IL-2-4D group, the IL-7R-IM group demonstrated upregulated activation of downstream signals, such as JAK/STAT5 transcription factors (*RUNX1* and *RUNX3*), markers of cell division (*CDK1)*, anti-apoptotic genes (*BCL2L1* and *BIRC5)*, cytotoxicity-related genes (*GZMA* and *GZMB*), and activating receptors (*NCR2* and *NCR3*). In contrast, genes associated with pro-apoptotic (*BAX*, *BAD* and *BBC3*) and inhibitory receptors (*KLRB1* and *LAG3*) were downregulated in the IL-7R-IM group (**Figure 4E**). In addition, the expression of inhibitory receptors *KLRD1* and *KLRC1* was upregulated in the IL-7R-IM group, suggesting that IL-7R-IM regulates NK cell function by balancing the expression of activating and inhibiting receptors. These differences further suggest that IL-7R-IM promotes the proliferation, viability, and cytotoxicity of NK92 cells. It was shown that NK92-IL-7R-IM is more effective than IL-2-maintained NK92 cells in maintaining cell viability and cytotoxicity by mediating the expression of downstream target genes (*GZMB*, *GZMA*, *BCL2L1*, *BAX*) of the JAK-STAT5 pathway (25). In summary, these data indicated that IL-7R-IM enhanced the viability and tumor-killing activity of NK92 cells by regulating the expression of IL-7R downstream target genes and receptors through sustained activation of the IL-7R downstream pathway.

### IL-7R-IM enhances the anti-tumor activity of NK92 cells *in vivo*

**Figure 5:**
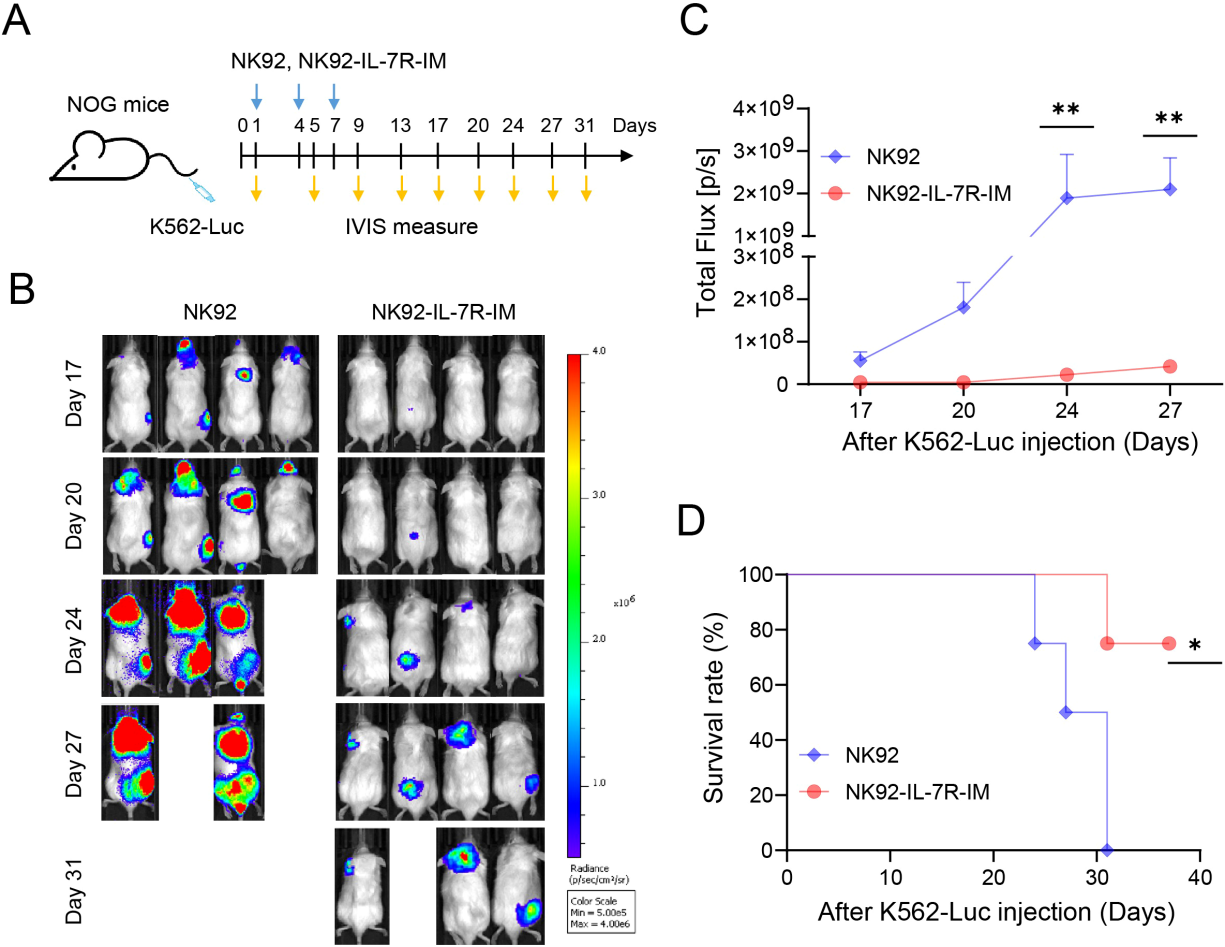
IL-7R-IM enhances the anti-tumor activity of NK92 cells *in vivo* **(A)** Mouse experimental scheme. **(B)** Images of the dorsal bioluminescence (BLI) following the injection of NK92 or NK92-IL-7R-IM cells. **(C)** Quantification and statistical analysis of the BLI. **(D)** Survival curves after the injection of K562-Luc cells. Data are represented as the mean ± SEM. Statistical significance was assessed by two-way ANOVA followed by Šidák’s multiple comparisons test. **(C)** or the Mantel-Cox test **(D)**. **p < 0.01; *p < 0.05. The animal studies were performed as two independent experiments showing consistent overall trends. The data shown represent one representative experiment (n = 4 mice per group).

Next, to test whether IL-7R-IM enhances the anti-tumor activity of NK92 cells *in vivo*, we constructed a leukemia model using luciferase-expressing K562 cells (K562-Luc). K562-Luc cells were injected firstly. The BLI results were measured one day after the injection of K562-Luc cells and reflected a relatively similar number of K562-Luc cells was implanted across each mouse, and the mice were then randomly divided into two groups. (**Supplementary Figure S3A**). Next, NK92 or NK92-IL-7R-IM cells were intravenously transfused thrice into NOG mice (**Figure 5A**). The results showed that mice injected with NK92-IL-7R-IM cells had lower tumor bioluminescence (**Figures 5B, C**) (*p* < 0.01) and higher survival rates than the mice injected with NK92 cells (**Figure 5D**) (*p* < 0.05). Therefore, IL-7R-IM enhanced the anti-tumor activity of NK92 cells against K562 leukemia *in vivo*, suggesting that constitutive IL-7R signaling can maintain NK cell activity and extend survival.

In addition, the *in-vivo* persistence of NK92 and NK92-IL-7R-IM cells was measured. Initially, we conducted an experiment that 2μg of luciferase-coding mRNA was electroporated into 2×10^6^ NK92 and NK92-IL-7R-IM cells, followed by injection into mice after 15 hours. Bioluminescence imaging (BLI) was performed using an IVIS machine at different time points to measure luciferase expression. The *in vivo* results revealed that NK92-IL-7R-IM cells exhibited significantly higher luciferase expression compared to NK92 cells from 24 hours post-injection (**Supplementary Figure S3B**), indicating prolonged persistence *in vivo*. However, we observed that luciferase signals diminished almost entirely by 48 hours. Consistent with previous study (12), we find that NK92 cells inherently do not persist very well *in vivo*, regardless of IL-7R-IM expression. Nevertheless, these findings demonstrate that within this limited survival window, NK92-IL-7R-IM cells persist relatively longer *in vivo* than unmodified NK92 cells, which correlates with their enhanced anti-tumor efficacy.

### IL-7R-IM improves the proliferation, viability, and function of EphA2-CAR-NK92, ZEGFR-CAR-NK92, and CD5-CAR-NK92 cells

**Figure 6:**
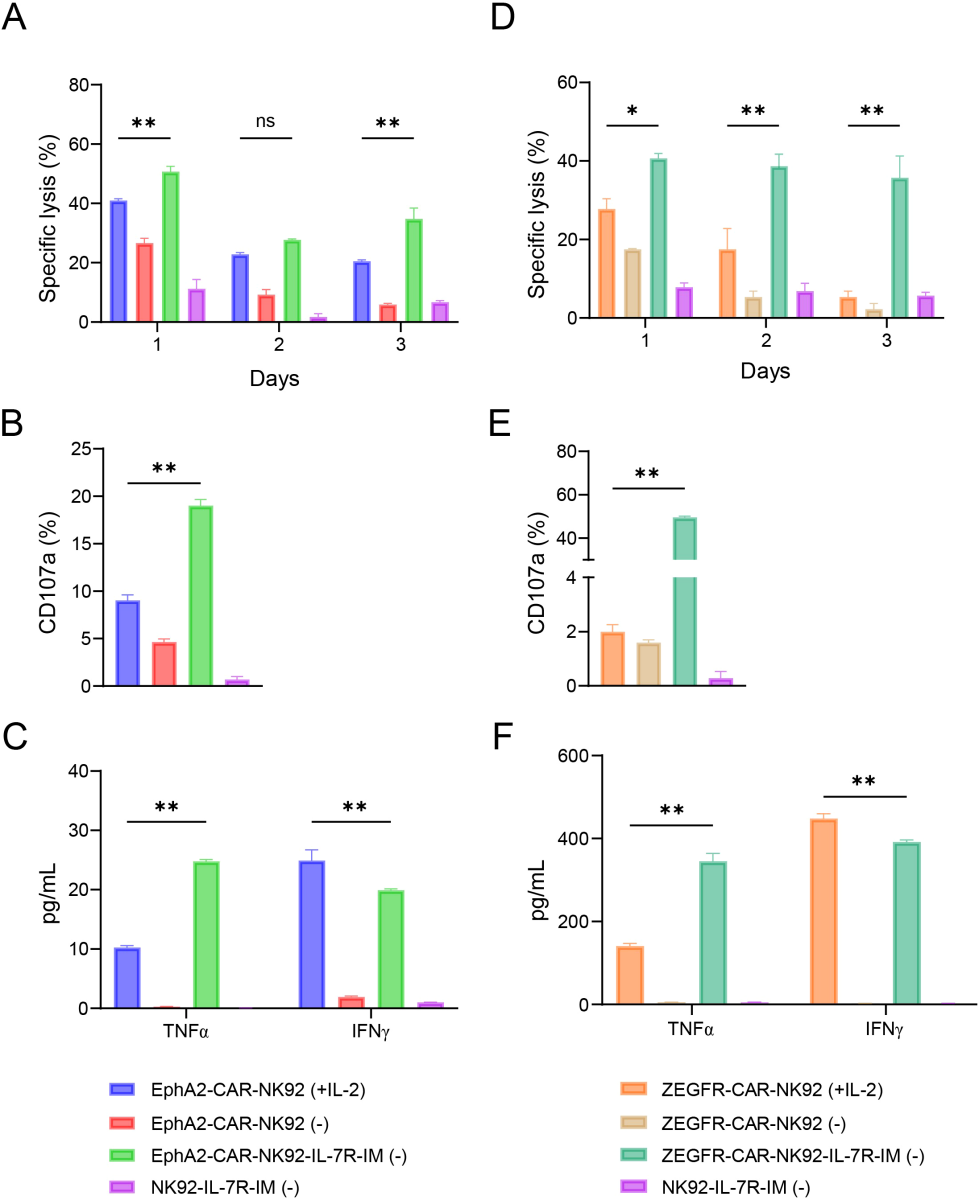
IL-7R-IM promotes the proliferation, viability and cytotoxicity of EphA2-CAR-NK92 and ZEGFR-CAR-NK92cells. In each experiments groups of EphA2-CAR-NK92 **(A-C)** and ZEGFR-CAR-NK92 **(B-D)**, cells were co-cultured with H460 target cells at an E:T ratio of 1:1. **(A, D)** Cytotoxicity was assessed after 1–3 days of culture. **(B, E)** Degranulation was analyzed by flow cytometry after 3 days of culture (with 4 h co-incubation). **(C, F)** The secretion of IFN-γ and TNF-α was determined by ELISA (after 12 h co-incubation). Data are presented as mean ± SEM. The treatment concentration of IL-2 is 20 ng/mL. Data represent the mean ± SEM of three independent experimental replicates. Statistical significance analyzed by two-way ANOVA followed by Tukey’s multiple comparisons test. **(A, C, D, F)** Statistical significance was assessed by ordinary one-way ANOVA. **(B, E)** **p < 0.01; *p < 0.05.

To investigate whether IL-7R-IM could enhance the anti-tumor efficacy of CAR-NK92 cells, we expressed IL-7R-IM in Ephrin type-A receptor 2-CAR-NK92 (EphA2-CAR-NK92) cells by lentiviral transduction. EphA2-CAR-NK92 cells can kill H460 and MDA-MB-231 cells (39). The procedure for obtaining EphA2-CAR-NK92-IL-7R-IM cells was the same as that for producing NK92-IL-7R-IM cells. Finally, IL-7R-IM expression levels in EphA2-CAR-NK92 cells were assessed via flow cytometry (**Supplementary Figure S4A**). We found that the EphA2-CAR-NK92-IL-7R-IM (–) group demonstrated increased cell proliferation than the EphA2-CAR-NK92 (+IL-2) group on day 3 of culture (**Supplementary Figure S4B**) (*p* < 0.01). In this study, we selected two target cells to verify the function of IL-7R-IM in CAR-NK92 cells. CAR-NK92 cells were co-cultured with each target cells to evaluate their cytotoxic potential. The cytolytic activity of the EphA2-CAR-NK92-IL-7R-IM (–) group was significantly higher than that of the EphA2-CAR-NK92 (+IL-2) group on days 1–3 of culture (**Figures 6A**; **Supplementary Figures S4C, R, F**) (*p* < 0.05, *p* < 0.01). Moreover, the degranulation activity (*p* < 0.01) (**Figure 6B** and **Supplementary Figures S4D**) and cytokine TNF-α release (*p* < 0.01) (**Figure 6C**) of EphA2-CAR-NK92-IL-7R-IM (–) group was significantly higher than that of the EphA2-CAR-NK92 (+IL-2) group, while cytokine IFN-γ release from the EphA2-CAR-NK92-IL-7R-IM (–) group was lower than that of the EphA2-CAR-NK92 (+IL-2) group at day 3 of culture. Altogether, these results suggest that constitutive IL-7R signaling was more effective than the addition of IL-2 in maintaining EphA2-CAR-NK92 cell viability and tumor killing activity.

To investigate whether IL-7R-IM has the potential to enhance the function of other CAR-NK92 cells, we expressed IL-7R-IM in epidermal growth factor receptor (EGFR) CAR-NK92 (ZEGFR-CAR-NK92) and CD5-CAR-NK92 cells. ZEGFR-CAR-NK92 cells have been shown to kill A549 lung cancer cells (40), and have been used as model cells for many CAR-NK92 experiments (41, 42). CD5-CAR-NK92 cells can be used to targeted acute lymphocytic MOLT4 cells (43). The procedure for obtaining ZEGFR-CAR-NK92-IL-7R-IM and CD5-CAR-NK92-IL-7R-IM cells was the same as that for producing NK92-IL-7R-IM cells. Finally, IL-7R-IM expression levels in ZEGFR-CAR-NK92 and CD5-CAR-NK92 cells were detected using flow cytometry (**Supplementary Figures S5A**, **S6A**). The results showed that the ZEGFR-CAR-NK92-IL-7R-IM (–) group demonstrated higher cell proliferation capacities than the ZEGFR-CAR-NK92 (+IL-2) group on day 3 of culture (**Supplementary Figure S5B**), whereas the CD5-CAR-NK92-IL-7R-IM (–) group showed the same cell growth as the CD5-CAR-NK92 (+IL-2) group on day 3 of culture (**Supplementary Figure S6B**). CD5-CAR-NK92-IL-7R-IM (–) groups was significantly higher than that of the CAR-NK92 (+IL-2) group on day 4 of culture (**Supplementary Figures S6C**) (*p* < 0.01). In addition, the cytolytic activity was enhanced in both IL-7R-IM edited CAR-NK92 cells (**Figure 6D**; **Supplementary Figures S6D**) (*p* < 0.01), degranulation activity (*p* < 0.05, *p* < 0.01) and cytokine release (IFN-γ and TNF-α) (*p* < 0.05, *p* < 0.01) of ZEGFR-CAR-NK92-IL-7R-IM were consistent with the trends observed in EphA2-CAR-NK92-IL-7R-IM cells (**Figures 6E, F**). Therefore, these results suggest that IL-7R-IM expression can promote cell viability and cytolytic activity in NK92 and a variety of CAR-NK92 cell lines, and is dependent on antigen activation.

## Discussion

 NK cell therapy has attracted considerable attention as an effective and safe novel immunotherapy (10). However, in the realm of adoptive NK cell immunotherapy, transplanted NK cells typically persist in the body for less than two weeks post-infusion (12, 16). Although the poor persistence of NK cells improves their safety, it hinders their therapeutic efficacy. Therefore, it is important to identify methods for improving the viability of NK cells to enhance their anti-tumor efficacy. Cytokines such as IL-2 and IL-15 can activate NK cells, augmenting the effectiveness of NK cell immunotherapy; however, the administration of IL-2 and/or IL-15 can cause serious toxicity. In addition, some methods have been developed to enable NK92 cells to express membrane-bound IL-2 to maintain the activity of NK92 (10, 12), but these are still limited by the number of receptors and affinity of ligands. Here, we developed a new strategy for expressing IL-7R-IM in NK92 and CAR-NK92 cells, allowing NK cells to maintain high cell viability and tumor-killing activity under cytokine-independent conditions, which will help improve the anti-tumor capacities of NK cells.

In the present study, we systematically explored the function of IL-7R in NK92 cells for the first time. To verify the function of IL-7R, we constructed NK92-IL-7R-WT cells by infecting NK92 cells with a WT IL-7R-carrying lentivirus. Consistent with our hypothesis, IL-7-IL-7R activation can promote NK92 cell proliferation, viability, and tumor-killing activity by promoting TNF-α and IFN-γ release. Previous studies have shown that IL-7 can promote the release of IFN-γ (28), which is consistent with our results. Our results also indicate that IL-7 can promote the release of TNF-α. After 1–2 days of cell culture, IL-2 treatment increased the tumor-killing activity and TNF-α and IFN-γ release from NK92 cells more than IL-7 treatment, demonstrating that IL-2 is also critical for NK92 cell activation. However, IL-7 treatment was stronger than IL-2 treatment in enhancing the proliferation, viability, and cytotoxicity of NK92 cells after 3–4 days of continuous culture. These results suggest that IL-7R has the potential to be used in NK cell immunotherapy to enhance its antitumor activity.

We further expressed IL-7R-IM in NK92 cells to reduce their cytokine dependence. IL-7R-IM can support the proliferation and survival of NK92 cells independently of exogenous cytokines and maintain cell viability and cytotoxicity better than the addition of IL-2. In addition, IL-7R-IM promoted the degranulation activity and cytokine (TNF-α and IFN-γ) release from NK92 cells to improve NK cell-mediated tumor-killing activity.

At present, in addition to the construction of NK cells via lentiviral transduction, mRNA transfection is also widely used. We transfected IL-7R-IM mRNA to improve pNK cells viability and cytotoxicity *in vitro*. IL-7R-IM has the same function in pNK and NK92 cells and can maintain cell viability and NK cell cytotoxicity in the absence of cytokines. We believe that IL-7R-IM mRNA has potential in future cancer therapies. Furthermore, it is important to note that while our *in vitro* data using mRNA electroporation convincingly demonstrate enhanced survival and function of primary NK cells during the peak expression window (12 hours), this acute evaluation does not establish sustained, long-term cytokine-independent survival. Therefore, further studies employing stable gene transfer methods in primary NK cells are required to fully validate long-term cytokine independence and persistence.

Other studies have used various strategies to enhance the anti-tumor effects of NK92 cells (44, 45). However, delivering IL-7 signals to NK cells via IL-7R-IM has distinct advantages. The unique advantage of our IL-7R-IM strategy lies in the 'ligand-independence' conferred by the insertion mutation. Unlike conventional approaches that rely on the overexpression of wild-type IL-7R (which still requires exogenous IL-7) or IL-7-secreting CARs (which depend on autocrine/paracrine loops and complex vector engineering), our IL-7R-IM system constitutively activates downstream signaling solely through a structural modification in the transmembrane domain. This distinction is critical in the solid tumor microenvironment (TME), which is characteristically an 'IL-7 desert.' As demonstrated by our comparative data between IL-7R-WT and IL-7R-IM (**Figures 1**, **2**), the IM-modified NK cells sustain robust viability, proliferation, and cytotoxicity even in the complete absence of cytokine support. This 'always-on' signaling capability makes our platform a more robust and versatile strategy for overcoming the cytokine-deprived conditions of the TME compared to strategies that remain contingent upon ligand availability. When compared with NK92 cell therapy in the presence of exogenous cytokines, IL-7R-IM caused NK92 cells to exhibit better cytolytic activity independently of cytokine levels and significantly reduced the treatment-related toxicity associated with cytokine administration. Recently, two new types of non-secretory IL-2 fusion proteins have been developed (10, 12), but IL-2 fusion efficiency remains limited by the number of intracellular IL-2Rα receptors. IL-7R-IM-based treatment overcomes these concerns.

IL-2 and IL-7 have been shown to share the same signaling factor in T cells (46), and IL-7 mediates STAT activation. Thus, IL-7 is essential for T cell proliferation, survival, and cytotoxicity (47). This study is the first to explore the signaling output of IL-7R-IM in NK92 cells. We found that IL-7R-IM consistently activated the JAK1-STAT5, AKT, and ERK signaling pathways, which was consistent with our hypothesis and previous studies on IL-7R insertion mutations (48). Therefore, our results underscore the role of IL-7 in mediating STAT5 activation, which is pivotal for the proliferation and survival of NK92 cells. These pathways, namely PI3K/AKT and MEK/ERK, are known to regulate cell proliferation (25). Previous studies have shown that BCL2 mediates the anti-apoptotic effect of IL-7 (49). Our gene expression analysis results showed that the promoting effect of IL-7R-IM on NK92 cell survival is associated with increased *BCL2L1* expression and decreased *BAX*/*BAD* expression. *BCL2L1, BAX*, and *BAD* are all members of the BCL-2 family. Moreover, upregulation of cytotoxicity-related *GZMA* gene expression has also been shown to be involved in the anti-tumor activity of IL-7 (35). The repertoire of NK cell receptors is critical for NK cell recognition by tumor cells (3), and the net balance of stimulatory and inhibitory signals through these receptors results in either a response or tolerance of the target cells (50). Cytokines such as IL-2 can upregulate the secretion of activating receptors and cytotoxic genes (12). Similarly, our results showed that the IL-7R-IM (–) group demonstrated upregulation of activating receptors (*NCR3 and NCR2*) and cytotoxicity-related genes (*GZMA and GZMB*), and downregulation of inhibitory receptors (*KLRB1* and *LAG3*) when compared with the NK92 (+IL-2) group after 4 days of culture. Overall, it was shown that NK92-IL-7R-IM cells possess superior anti-tumor killing abilities than NK92 (+IL-2) cells. Therefore, our results suggest that IL-7R-IM can promote the NK92 response to target cells and enhance their tumor-killing activity not only by upregulating degranulation activity and cytokine (TNF-α) release, but also by upregulating activating receptors (*NCR2* and *NCR3*) and cytotoxicity-related genes (*GZMA* and *GZMB*) in NK92 cells.

To further verify the effect of IL-7R-IM, we constructed an animal model of leukemia and showed that NK92-IL-7R-IM cells demonstrated superior tumor growth inhibition and higher survival rates than NK92 cells, underlining the effect of IL-7R-IM. The core mechanism of NK cell therapy is not to achieve long-term survival and memory responses like T cells, but to reduce the tumor burden with a powerful initial burst of killing power immediately after administration. This slows tumor growth and delays tumor progression. Human NK92 cells, when injected into mice, are known to rapidly clear from the bloodstream and accumulate in tissues, with a survival duration of approximately 24 to 72 hours (12, 51). In our K562 leukemia mouse model, the therapeutic efficacy of NK92 cells is evaluated not by completely eradicating leukemia, but by measuring their ability to slow the progression of the disease. This approach reflects the transient yet impactful role of NK92 cell therapy in suppressing tumor growth during their limited survival *in vivo*. Although NK92 cells survived for only 1 day as detected by the luciferase method, NK92 served as a control cell, which started killing tumor cells and controlling the number of tumor cells immediately after injection. Given this context, the prolonged antitumor activity observed with NK92-IL-7R-IM cells can be attributed to their extended persistence compared to unmodified NK92 cells. The extended survival of NK92-IL-7R-IM cells allows for a more sustained therapeutic effect, effectively delaying the progression of leukemia. This highlights the enhanced bioavailability and functional longevity of NK92-IL-7R-IM cells as a critical factor in their superior antitumor efficacy.

IL-7 has been used in immunotherapy studies on CAR-T cells because of its crucial role in T cell proliferation (25). An engineered IL-7R insertion mutation promotes survival, proliferation, and the anti-tumor activity of CAR-T cells (35). Thus, IL-7R-IM can be considered to enhance the immunotherapeutic efficacy of CAR-NK cells. Therefore, IL-7R-IM was expressed in EphA2-CAR-NK92, ZEGFR-CAR-NK92, and CD5-CAR-NK92 cells to verify its utility in CAR-NK92 therapy. As expected, *in vitro* experiments showed that IL-7R-IM promoted the cell survival and cytotoxicity in these three CAR-NK92 cell lines when compared with normal CAR-NK92 cells. While CAR-NK cells have demonstrated effectiveness in combatting hematologic malignancies, its application in the treatment of solid tumors remains limited by certain challenges (52). IL-7R-IM-expressing is expected to promoted anti-tumor capacity of ordinary CAR-NK cells, providing support for its utility in addressing current issues in NK cell immunotherapy for the treatment of solid tumors.

Our findings align with and expand upon recent advancements in cytokine engineering. Previous studies demonstrated the potential of constitutively active IL-7R (C7R) in enhancing T-cell therapies (35). Furthermore, a very recent study reported that C7R signaling significantly promotes the survival of CAR-NK cells (53), which is consistent with our observation that IL-7R-IM enhances NK cell persistence. While Dysthe et al. noted that C7R-expressing CAR-NK cells exhibited moderate *in vivo* efficacy compared to IL-15-activated counterparts, they still demonstrated superior therapeutic effects over conventional CAR-NK cells. This is consistent with our *in vivo* findings in NK92 cells, where IL-7R-IM conferred a significant survival and anti-tumor advantage. Importantly, our study complements these findings by demonstrating the versatile applicability of IL-7R-IM signaling across CAR-NK92 cells targeting diverse antigens (EphA2, EGFR, and CD5). Collectively, these studies underscore the critical impact of continuous IL-7 signal transduction on NK and CAR-NK cell fitness, providing a valuable reference for future cytokine design strategies aimed at enhancing the persistence and function of adoptive cell therapies.

In this study, a comparison of IL-2 cytokine signaling with IL-7-induced NK92 cell cytotoxic activity demonstrated that IL-7 exhibits greater durability in sustaining NK cell activity. IL-7R-IM-NK cells, engineered with insertion mutations, were introduced to address NK cell cytokine dependency. Compared with conventional methods that use IL-2, NK92-IL-7R-IM cells have higher anti-tumor activity with fewer adverse reactions. Furthermore, the limitation of membrane-bound IL-2 or cytokine receptor expression—constrained by receptor number and affinity—was addressed. IL-7R-IM enhanced NK cell activation without requiring exogenous cytokines.

In conclusion, IL-7R-IM maintained cell survival and the cytotoxicity of NK92 and CAR-NK92 cells independently of cytokine levels, and IL-7R-IM improved the efficacy of NK92 against liquid tumors. Therefore, we developed a strategy that enables IL-7R-IM-expressing NK92cells to maintain highly effective anti-tumor activity in the tumor microenvironment. We believe that IL-7R-IM has promising future applications in immunotherapy. Additionally, our study did not employ primary NK cells for *in vivo* experiments, which is another limitation of our approach. Primary NK cells are more representative of clinical settings, but their limited viability and the challenges associated with their genetic modification posed significant experimental barriers. Addressing these limitations, particularly through the use of primary NK cells in future studies, will be critical for enhancing the translational relevance of our findings. Future studies will elucidate IL-7 signaling mechanisms, differentiate iPSCs into NK cells, and establish stable IL-7R-IM expression to overcome gene-editing limitations in primary cells. The regulatory role of IL-7R in NK cell function will be explored to provide deeper insights into its mechanisms. Further investigation of the molecular mechanisms underlying IL-7R-regulated NK cell function will lay a foundation for optimizing NK cell-based therapies.

## Data Availability

The data presented in the study are deposited in the NCBI Gene Expression Omnibus (GEO) repository, accession number GSE246165.
