# Supplementary material for: IL-7Rα signaling potentiates the anti-tumor activity of NK92 cells
Source: Front Immunol. 2026 Mar 30;17:1768539. doi: 10.3389/fimmu.2026.1768539 (PMC13071061; doi:10.3389/fimmu.2026.1768539)
Supplement: Supplementary file 1 [file DataSheet1.pdf]

Supplemental information

IL-7Rα signaling potentiates the anti-tumor activity of NK92 cells

Chunli Wang[1,2,#], Seokmin Kim[1,3,#], Ling-Zu Kong[1,4,#], Inhwan Jang[1], Seona Jo [1,5], Sunyoung Lee [1], Soo Yun Lee[1], Kee K. Kim[4], Tae-Don Kim[1,3,5,*]

[1]Center for Gene and Cell Therapy, Korea Research Institute of Bioscience and Biotechnology (KRIBB), Daejeon 34141, Republic of Korea

[2]Key Laboratory of Laboratory Medicine, Ministry of Education, School of Laboratory Medicine and Life Sciences, Wenzhou Medical University, Wenzhou, Zhejiang 325000, PR China

[3]Department of Pharmacy, Yonsei Institute of Pharmaceutical Sciences, and Department of Integrative Biotechnology, College of Pharmacy, Yonsei University, Incheon 21983, Republic of Korea

[4]Department of Biochemistry, College of Natural Sciences, Chungnam National University, Daejeon 34134, Republic of Korea

[5]KRIBB School of Advanced Bioconvergence, University of Science and Technology (UST), Daejeon 34113, Republic of Korea

*Correspondence, tdkim@kribb.re.kr (T.D.K)

[#]These authors contributed equally

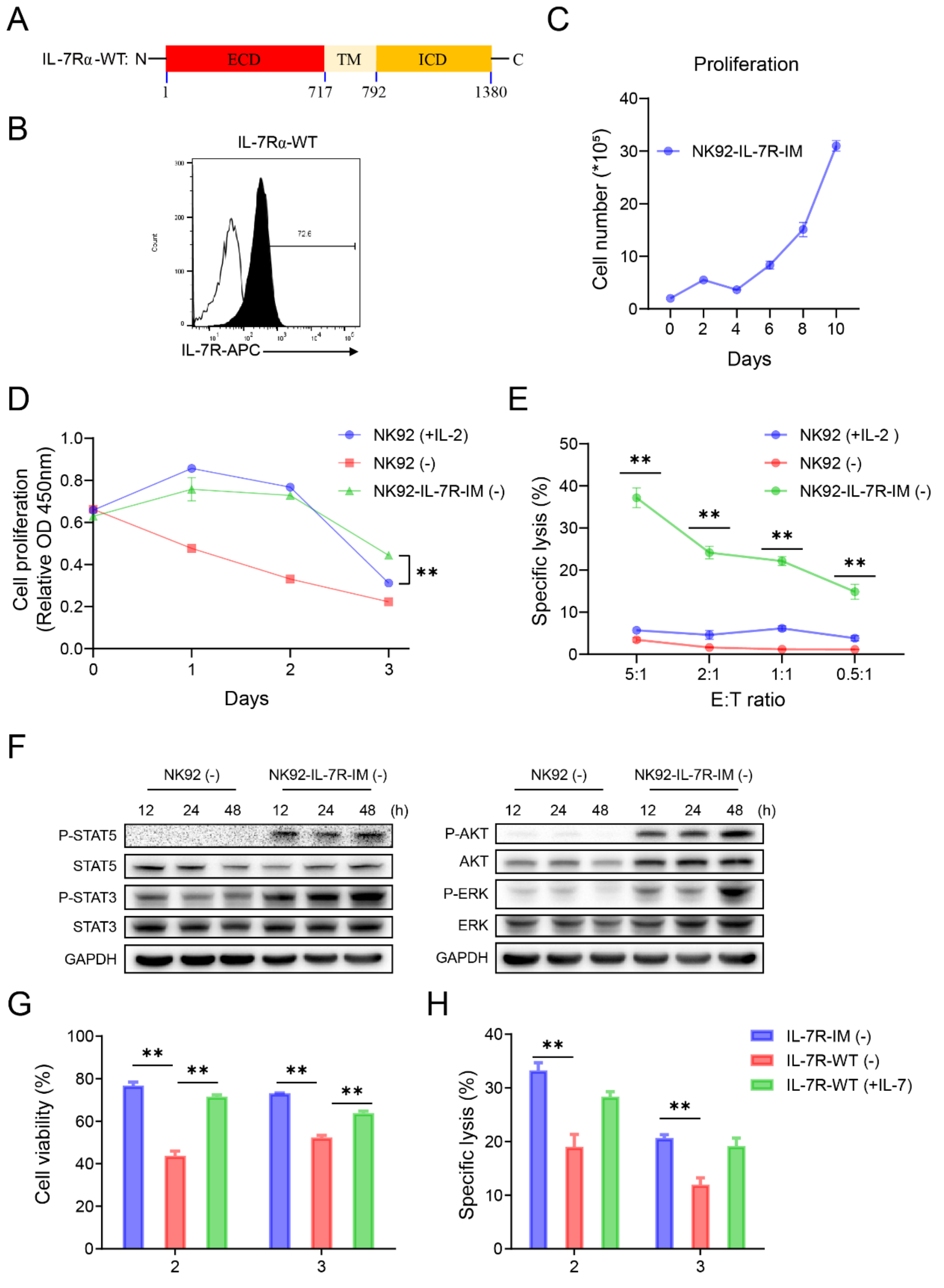

Figure S1. IL-7R-WT and IL-7R-IM edited NK92 cells and pNK cells

(A) Schematic representation of IL-7R wild type expression cassette. ICD: intracellular domain, TM: transmembrane region, ECD: extracellular domain. (B) Representative flow cytometry detects showing the expression of IL-7R-WT in NK92 cells. (C) NK92-IL-7R-IM expansion rate under the cytokine free condition. (D) CCK-8 proliferation assay results of NK92 maintained with IL-2 (20 ng/mL) (+IL-2) or without cytokines (-) and NK92-IL-7R-IM cells

maintained without cytokines (-) after being cultured for 1-3 days. (E) Cytotoxicity against K562 cells, mediated by the three groups of cells cultured for 3 days, E: T ratios=5:1, 2:1, 1:1, and 0.5:1. (F) Western blot evaluation of STAT5, STAT3, ERK, and AKT kinase phosphorylation level in NK92 and NK92-IL-7R-IM cells maintained without cytokines for 12, 24, and 48 hours. (G) Cell viabilities of the pNK cells were measured 12 h after electroporation of 10 μg mRNA. (H) Cytotoxic activities of the pNK cells were detected 12h after electroporation of 10 μg mRNA. (G-H) A total of three donors were included in this experiment. The labels "2" and "3" on the x-axis represent the remaining two donors. Data represent the mean ± SEM of three independent experimental replicates. Statistical significance was assessed by an unpaired, two-tailed Student's t-test. $^{**}p < 0.01$.

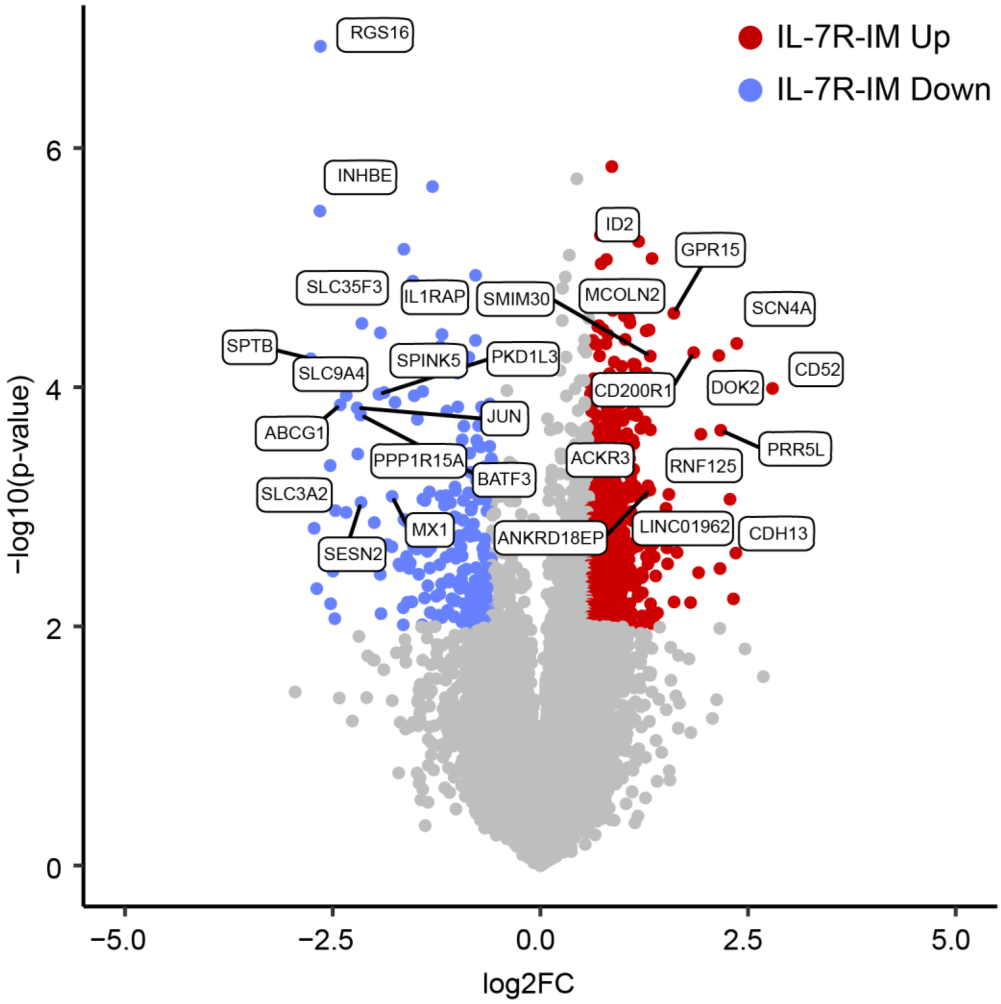

Figure S2. The differentially expressed proteins analyzed by volcano plots between IM-4D, IL-2-2D and IL-2-4D groups.

Dark dots represent genes that are not significantly differentially expressed (fold change < 1.5, p < 0.01), red dots represent significantly upregulated genes, and green dots represent significantly downregulated genes.

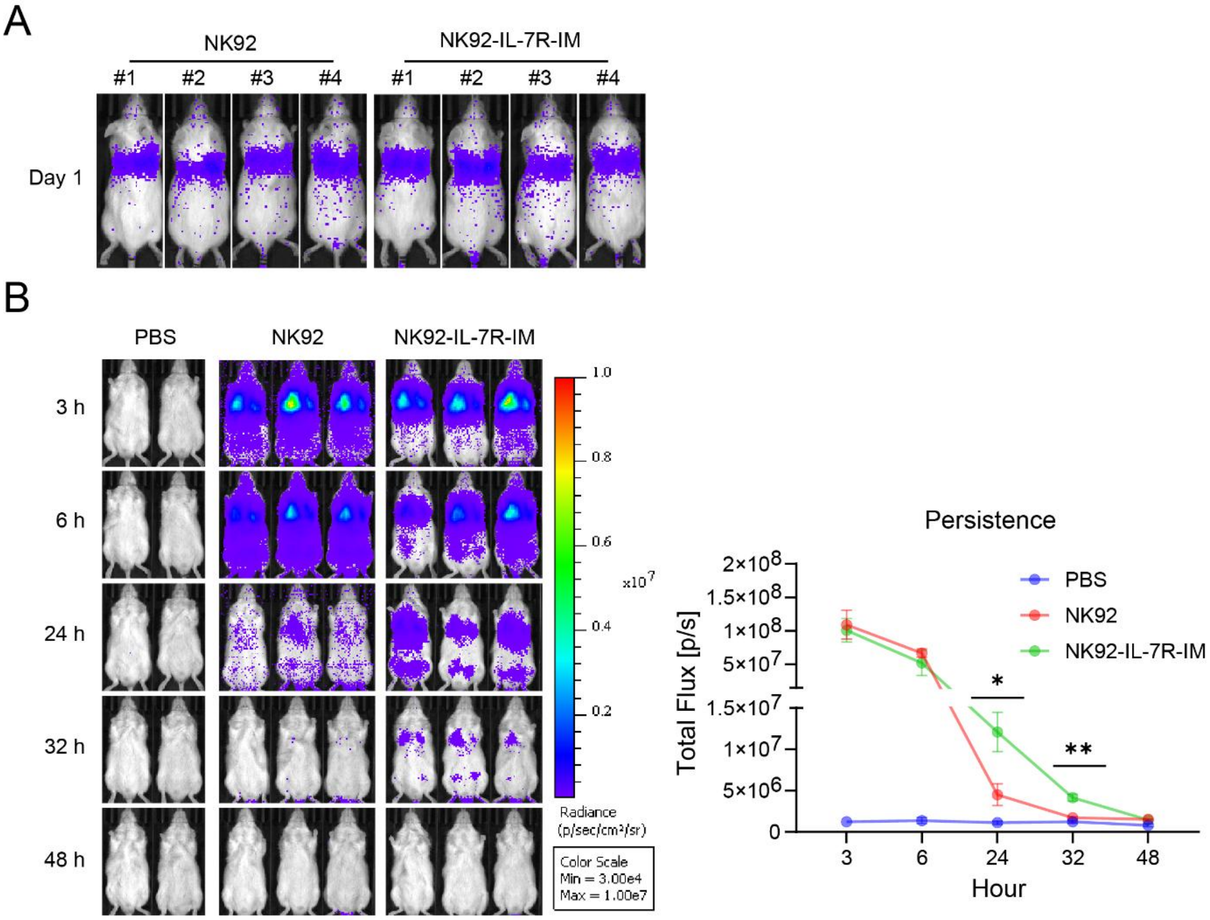

Figure S3. The in-vivo persistence of NK92 and NK92-IL-7R-IM cells.

(A) Images of the bioluminescence (BLI) following the injection of NK92 or NK92-IL-7R-IM cells (n = 4). (B) Images of the bioluminescence following the injection of NK92 or NK92-IL-7R-IM cells (n = 3), Quantitative analysis of total fluorescence is shown. The animal studies were performed as two independent experiments showing consistent overall trends. The data shown represent one representative experiment. Data are represented as mean ± SEM. Statistical significance was assessed by an unpaired, two-tailed Student's t-test. $^{**}p < 0.01$; $^{*}p < 0.05$.

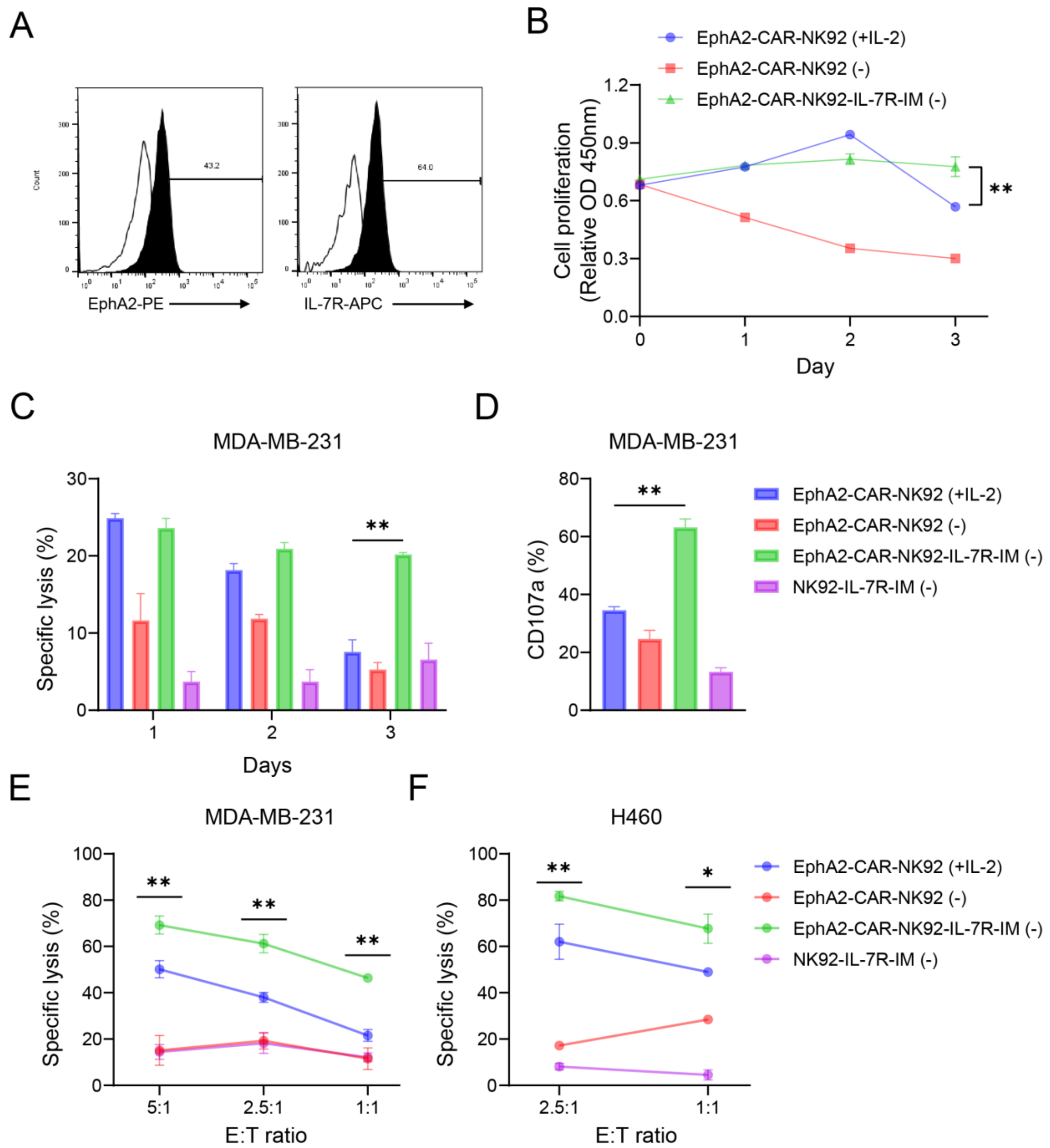

Figure S4. IL-7R-IM promotes the proliferation, viability and cytotoxicity of EphA2-CAR-NK92 cells.

(A) Flow cytometry detects showing the expression of IL-7R-IM in EphA2-CAR-NK92 cells. (B) CCK-8 proliferation assay of EphA2-CAR-NK92 maintained with IL-2 (+IL-2) or without cytokine (-) and EphA2-CAR-IL-7R-IM cells maintained without cytokine (-). (C) Cytotoxicity against MDA-MB-231 and H460 cells mediated by four group cells cultured for 1-3 days, E:T ratio=1:1. Data are represented as mean ± SEM. (D) Degranulation was detected in NK92 cells cultured for 3 days by flow cytometry after co-incubated with MDA-MB-231 and H460 cells at the E:T ratio of 1:1. (E-F) Cytotoxicity against MDA-MB-231 and H460 cells mediated by four group cells cultured for 3 days, E: T ratios=5:1, 2.5:1, 1:1. Data represent the mean ± SEM of three independent experimental replicates. Statistical significance was

assessed by an unpaired, two-tailed Student's t-test. **$p < 0.01$.

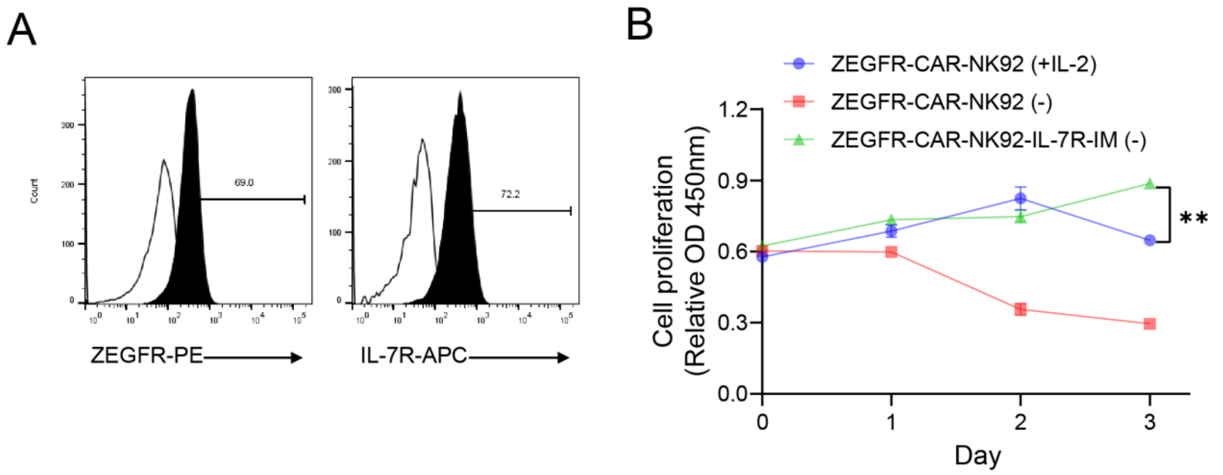

Figure S5. IL-7R-IM promotes the proliferation of ZEGFR-CAR-NK92 cells

(A) Flow cytometry detects showing the expression of IL-7R-IM in ZEGFR-CAR-NK92 cells. (B) CCK-8 proliferation assay results of ZEGFR-CAR-NK92 cells maintained with IL-2 (+IL-2) or without cytokines (-) and ZEGFR-CAR-IL-7R-IM cells maintained without cytokines (-) during 1-3 days of culture. Data represent the mean ± SEM of three independent experimental replicates. Statistical significance was assessed by an unpaired, two-tailed Student's t-test. **$p < 0.01$.

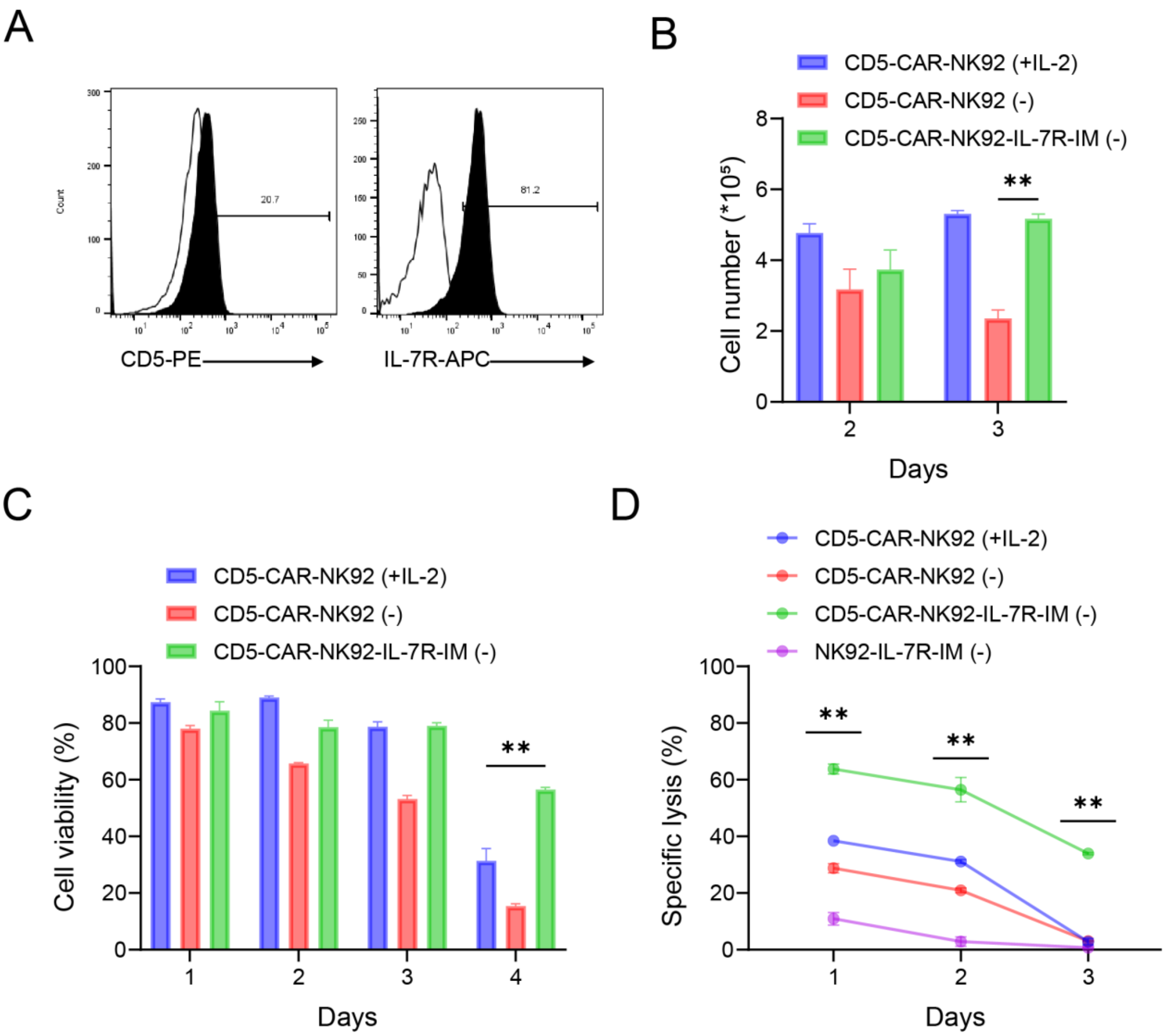

Figure S6. IL-7R-IM promotes the proliferation, viability and cytotoxicity of CD5-CAR-NK92-IL-7R-IM cells.

(A) Flow cytometry detects showing the expression of IL-7R-IM in CD5-CAR-NK92 cells. (B) Expansion of CD5-CAR-NK92 maintained with IL-2 (+IL-2) or without cytokine and CD5-CAR-IL-7R-IM maintained without cytokine. (C) The cell viability of CD5-CAR-NK92 (+IL-2), CD5-CAR-NK92 (-), and CD5-CAR-IL-7R-IM (-) cells. (D) Cytotoxicity against MOLT4 cells mediated by four group cells were cultured for 1-3 days, E: T ratio=2:1. Data represent the mean ± SEM of three independent experimental replicates. Statistical significance was assessed by an unpaired, two-tailed Student's t-test. ***p* < 0.01.

Table S1. Primer information of IL-7R-WT and IL-7R-IM used for constructing lentivirus vectors

F: CCGGAATTCGCCACCATGACAATTCTAGGTACAACTTTTGG
R: ATTTGCGGCCGCTCACTGGTTTTGGTAGAAGCTG
R1: TGGTGGGACAAGTTAGTAAGATAGGATCCATCTCCC
F1: CTTGTCCCACCATCAGCATTTTGAGTTTTTTCTCTG
